# Asprosin Enhances Cytokine Production by a Co-Culture of Fully Differentiated Mature Adipocytes and Macrophages Leading to the Exacerbation of the Condition Typical of Obesity-Related Inflammation

**DOI:** 10.3390/ijms24065745

**Published:** 2023-03-17

**Authors:** Agnieszka I. Mazur-Bialy

**Affiliations:** Department of Biomechanics and Kinesiology, Faculty of Health Science, Jagiellonian University Medical College, Skawińska 8, 31-066 Krakow, Poland; agnieszka.mazur@uj.edu.pl; Tel.: +48-012-421-9351

**Keywords:** asprosin, inflammation, obesity, obesity-related inflammation, macrophages, adipocytes

## Abstract

Asprosin, a fasting-induced, glucogenic, and orexigenic adipokine, has gained popularity in recent years as a potential target in the fight against obesity and its complications. However, the contribution of asprosin to the development of moderate obesity-related inflammation remains still unknown. The present study aimed to evaluate the effect of asprosin on the inflammatory activation of adipocyte–macrophage co-cultures at various stages of differentiation. The study was performed on co-cultures of the murine 3T3L1 adipocyte and the RAW264.7 macrophage cell lines treated with asprosin before, during, and after 3T3L1 cell differentiation, with or without lipopolysaccharide (LPS) stimulation. Cell viability, overall cell activity, and the expression and release of key inflammatory cytokines were analyzed. In the concentration range of 50–100 nM, asprosin increased the pro-inflammatory activity in the mature co-culture and enhanced the expression and release of tumor necrosis factor α (TNF-α), high-mobility group box protein 1 (HMGB1), and interleukin 6 (IL-6). Macrophage migration was also increased, which could be related to the upregulated expression and release of monocyte chemoattractant protein-1 (MCP-1) by the adipocytes. In summary, asprosin exerted a pro-inflammatory effect on the mature adipocyte–macrophage co-culture and may contribute to the spread of moderate obesity-associated inflammation. Nevertheless, further research is needed to fully elucidate this process.

## 1. Introduction

Asprosin, discovered in 2016 by Romere et al., is an adipokine released mainly by adipocytes in the white adipose tissue during fasting conditions [1]. Its primary function is the regulation of glucose production and its release from the liver, and hence, it is considered a glucogenic peptide [1,2]. Moreover, Li et al. [3] reported that glucose production stimulated by asprosin is also observed in diet-induced obesity. This recent study also showed that asprosin is involved directly in stimulating food intake. After crossing the blood–brain barrier, it activates agouti-related protein (AgRP) neurons in the hypothalamus as an orexigenic peptide [2]. Undoubtedly, under normal conditions, this molecule activity contributes to maintaining the proper energy homeostasis by the regulation of appetite and glucose secretion. On the other hand, some studies have shown that its pathologically elevated level is observed in obese individuals [4,5] as well as in patients with insulin resistance [6,7] and diabetes mellitus type 1 (DM1) [8] or type 2 (DM2) [9,10,11]. The use of anti-asprosin antibodies reduced food consumption by attenuation of AgRP neurons activation in obese individuals and potentially may contribute to combat obesity and related diseases [1,12]. Although asprosin is a newly discovered molecule, the effects of its action are relatively well known due to the high interest in research on this protein; for a review, see [13]. Nevertheless, still little is known about its mechanism of action, especially in terms of mechanisms related to the induction of inflammation in crucial insulin-sensitive tissues (skeletal muscle and pancreatic beta cells) and the enhancement of tissue damage by activating pro-inflammatory mechanisms [14]. Asprosin has been shown to impair insulin secretion from pancreatic beta cells under hyperglycemic condition by activating pro-inflammatory mechanisms related to the TLR4 pathway [15]. It was also found that asprosin reduces skeletal muscle insulin sensitivity by generating ER stress and inducing pro-inflammatory factors [16], as well as potentiates hyperlipidemia-induced endothelial inflammation in obese individuals by activating the IKKβ-NF-κBp65 pathway [17]. However, to date, there is no evidence that asprosin is directly or indirectly responsible for modulating adipocyte function resulting in obesity-related inflammation. Nevertheless, its significantly elevated level in obesity and the related enhancement of tissue damage, as well as the activation of a proinflammatory response in the pancreas and skeletal muscle suggest that it is, at least in part, involved in the regulation of moderate obesity-related inflammation. Therefore, the present study aimed to determine whether asprosin has a modulating effect on the course of moderate inflammation in an adipocyte–macrophage co-culture system which mimics the state of obesity related-inflammation. It is also important to determine whether the stage of adipocyte maturity, i.e., the hypertrophy of the adipocytes, affects the nature of asprosin action.

## 2. Results

### 2.1. Overall Activity and Viability of the Cell Co-Culture

The first stage in this research was the evaluation of the effect of asprosin on the overall viability and activity of cell co-cultures at different stages of differentiation. The evaluation was performed on the 7th, 14th, and 21st day of differentiation, wherein on day 21, a fully differentiated and hypertrophic adipocyte culture was obtained, corresponding to the hypertrophied adipocytes observed in obese individuals.

**Figure 1:**
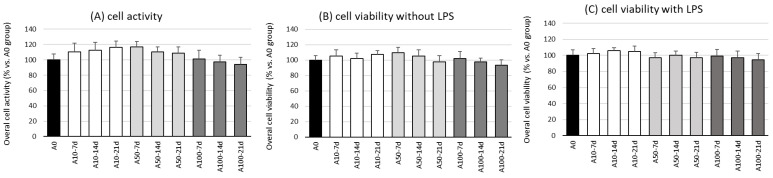
Effects of asprosin on overall cell activity measured in a CellTiter test (**A**) and viability (**B**) of adipocyte–macrophage co-cultures after 7, 14, and 21 days of differentiation, with lipopolysaccharide (LPS) or (**C**) without LPS (100 ng/mL) stimulation. The results from five independent experiments are expressed as mean + standard deviation (SD). Statistical significance (*p* < 0.05) was determined by the Duncan *t*-test.

The studies showed no effect of asprosin (0–100 nM) on the viability and overall activity of the cells on the 7th, 14th, and 21st day of differentiation (Figure 1). Additional analysis, not reported here, indicated that the highest dose of asprosin (150 nM) reduced the viability of the fully differentiated adipocytes (day 21). Due to the cytotoxic effect of the high dose of asprosin (150 nM), this dose was omitted in further studies. Studies on the mechanism of the cytotoxic action of high doses of asprosin are in progress.

### 2.2. Asprosin Enhances Cytokine Production by the Co-Cultured Cells

In the next stage of the study, the effect of asprosin on the intensity of the expression and release of key proinflammatory cytokines such as TNF-α (tumor necrosis factor α), IL-1β, IL-6 (interleukin 1 and 6), and HMGB1 (high-mobility group box protein 1) was evaluated in co-cultures with and without lipopolysaccharide (LPS) stimulation. Studies have shown no effect of asprosin alone on the expression and release of the analyzed cytokines. The effect was observed only when LPS stimulation was carried out. In incompletely mature cell (LPS-stimulated) co-cultures (day 14), which are characterized by a lower saturation of lipid droplets [18], asprosin did not modulate the expression and release of key inflammatory cytokines. However, it was noted that at higher concentrations of asprosin, in low-maturity co-cultures (day 7), the intensity of expression and release of the main pro-inflammatory cytokines, i.e., TNF-α, IL-6, and HMGB1, were reduced as compared to those in the LPS-stimulated co-culture (*p* < 0.05).

**Figure 2:**
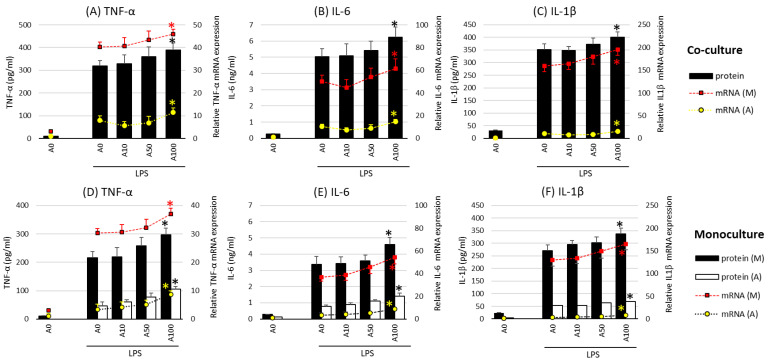
Effects of asprosin on lipopolysaccharide (LPS; 100 ng/mL)-induced cytokine mRNA expression and release: tumor necrosis factor-alpha (TNF-α) (**A**), interleukin-6 (IL-6) (**B**), and interleukin-1 beta (IL-1β) (**C**) in adipocyte–macrophage cocultures (upper panel) and adipocyte and macrophage monocultures (lower panel) for TNF-α (**D**), IL-6 (**E**), and IL-1β (**F**)on the 21st day of differentiation. The results are expressed as mean + standard deviation (SD) from five independent experiments. Statistical significance (*p* < 0.05) was determined by the Duncan *t*-test. The asterisk (*) above the bar denotes statistically significant differences in protein or mRNA levels, calculated relative to the A0 + LPS control group. Cells: (M) macrophages; (A) adipocytes.

**Figure 3:**
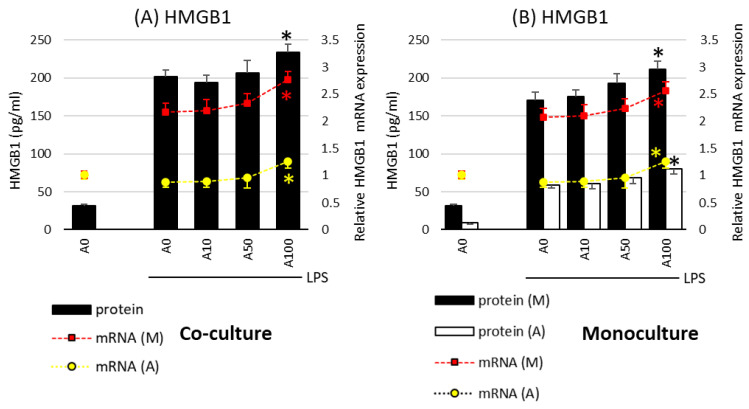
Effects of asprosin on lipopolysaccharide (LPS; 100 ng/mL)-induced cytokine mRNA expression and release of high-mobility group box-1 protein (HMGB1) in adipocyte “A” and macrophage “M” monocultures (**A**) and adipocyte–macrophage cocultures (**B**) on the 21st day of differentiation. The results are expressed as mean + standard deviation (SD) from five independent experiments. Statistical significance (*p* < 0.05) was determined by the Duncan *t*-test. The asterisk (*) above the bar denotes statistically significant differences in protein or mRNA levels, calculated relative to the A0 + LPS control group. Cells: (M) macrophages; (A) adipocytes.

A different effect of asprosin was observed when it acted on an LPS-stimulated co-culture containing fully differentiated and mature adipocytes (day 21). The highest concentration of asprosin (100 nM) resulted in the increased release of pro-inflammatory TNF-α, IL-6, IL-1β (Figure 2; *p* < 0.05), and HMGB1cytokines (Figure 3; *p* < 0.05). The analysis of these cytokines expression at the mRNA level showed that the increase in cytokine release was dependent on the enhancement of the expression of these cytokines in both macrophages and adipocytes (Figure 2, respectively). It should also be noted that the effect of asprosin seemed to be more pronounced in macrophages than in adipocytes. Moreover, the level of cytokine expression in macrophages was much higher than that in adipocytes. It was also noted that the expression of pro-inflammatory factors was significantly higher in adipocyte–macrophage co-cultures than in monocultures of adipocytes and macrophages, which suggests a significant additive effect of the cooperation of these cells in co-culture. These observations confirmed that both cell populations that build the adipose tissue and participate in the development of obesity-related inflammation are sensitive to the effects of asprosin, which acts on them as a pro-inflammatory activator.

### 2.3. Asprosin Influences the Release of the Key Adipokines Leptin and Adiponectin

**Figure 4:**
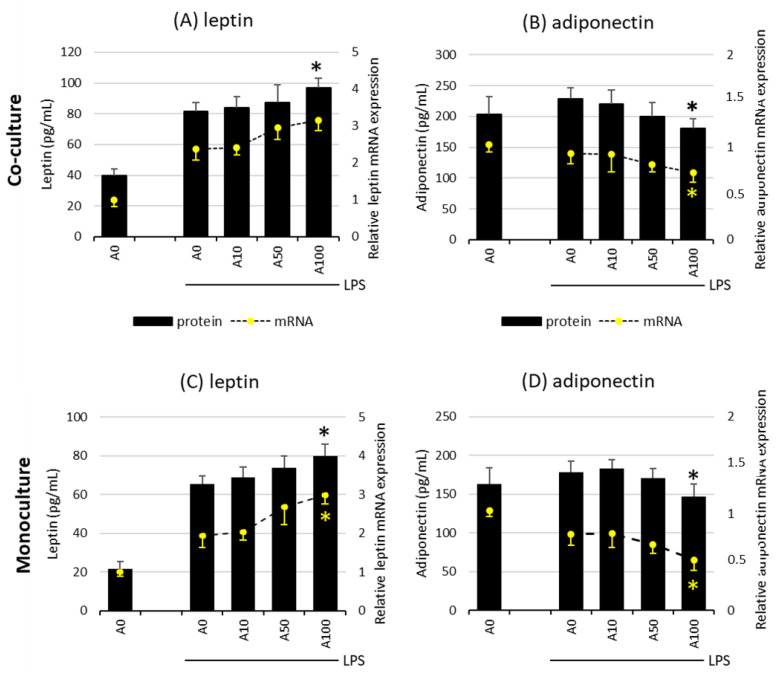
Effects of asprosin on LPS (100 ng/mL)-induced leptin expression and release (**A**) or adiponectin expression and release (**B**) by adipocyte–macrophage cocultures at 21 days of differentiation and leptin expression and release (**C**) or adiponectin expression and release (**D**) by adipocyte monocultures at 21 days of differentiation. 3T3 L1 adipocytes and RAW 264.7 macrophages were cultured in 12-well dishes with inserts (0.4 µm pore size) for 6 h (mRNA expression, rt-PCR) or 24 h (adipokine release, ELISA tests) with or without asprosin (0, 10, 50, or 100 nM; A0, A10, A50, or A100, respectively). The results are expressed as mean + standard deviation (SD) from five independent experiments. Statistical significance (*p* < 0.05) was determined by the Duncan *t*-test. The asterisk (*) above the bar denotes statistically significant differences in protein or mRNA levels, calculated relative to the A0 + LPS control group.

The effect of asprosin on the release of leptin and adiponectin, the key adipokines, depends on the degree of adipocyte maturity. There was no evidence of an effect of asprosin on the release of both adipokines in co-cultures on days 7 and 14, both at the mRNA and at the protein level, regardless of LPS stimulation. However, it was noted that the high concentration of asprosin acting on both the mature LPS-stimulated co-culture and adipocyte monoculture decreased the release of anti-inflammatory adiponectin with a simultaneous slight increase in the release of leptin. This effect was apparent at both mRNA and protein levels for adiponectin (Figure 4). As in the case of the release of key pro-inflammatory cytokines, the level of the analyzed adipokines was lower in the monoculture of adipocytes than in the co-culture, which may indicate the additive interaction of macrophages. The effect of asprosin itself on the level of the released adipokines by non-LPS-treated cells was not observed; the levels of both leptin and adiponectin were similar to those in control cells.

### 2.4. Asprosin Increases the Migration of Macrophages towards Cultured Mature Hypertrophic Adipocytes

The last stage of the study was the assessment of the influence of asprosin on the influx of macrophages into adipose tissue, resulting from the activity of chemotactic factors released by cells present in the adipose tissue. It is well known that macrophages are responsible for the development of moderate inflammation associated with obesity. For this purpose, the migration of macrophages into the supernatants of the adipocyte culture was investigated.

**Figure 5:**
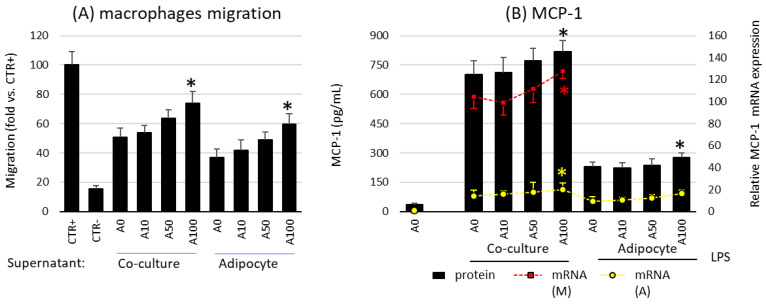
Effects of asprosin on (**A**) the migration of RAW 264.7 macrophages to the conditioned medium collected from co-culture or adipocytes cultured in the presence of various asprosin concentrations (0, 10, 50, or 100 nM) for 24 h (after 21 days of differentiation). Normal medium was used as a negative control (CTR−); N-formylmethionyl-leucyl-phenylalanine (fMLP) was used as a positive control (CTR+). (**B**) The effects of asprosin on lipopolysaccharide (LPS)-induced monocyte chemotactic protein 1 (MCP-1) mRNA expression and release by adipocyte–macrophage cocultures or adipocytes. The results are expressed as mean + standard deviation (SD) from five independent experiments. Statistical significance (*p* < 0.05) was determined by the Duncan *t*-test. The asterisk (*) above the bar denotes statistically significant differences in migration, protein or mRNA levels, calculated relative to the A0 (A) or A0 + LPS (100 ng/mL) control group. Cells: (M) macrophages; (A) adipocytes.

Studies have shown that asprosin influences the profile of released chemotactic factors and the migration of macrophages by acting on mature adipocyte cultures. A high dose of asprosin significantly increased the degree of macrophage migration toward both the co-culture and the adipocyte supernatant from cultures on day 21, but not from cultures on day 7 or day 14 (Figure 5A). This increase in migratory activity corresponded to an increase in both expression and release of the main macrophage chemotactic factor MCP-1 (Figure 5B). This effect was noted in cultures grown for 21 days but not in cultures grown for 7 or 14 days. In the case of cultures on day 7, a slight decrease in MCP-1 expression was noted, which, however, was not confirmed by a decrease in the release of the chemokine itself or a decrease in macrophage influx. Moreover, as noted for the previously mentioned cytokines, the expression of MCP-1 was significantly higher in macrophages than in adipocytes (Figure 5B). Thus, asprosin had a stronger effect on macrophages than on adipocytes. Nevertheless, the effect of asprosin itself on both macrophages influx and the level of released MCP-1 by non LPS-treated cells was not observed. It is likely that in the obesity state, its elevated level may increase the influx of immunocompetent cells into the adipose tissue, thereby increasing the pro-inflammatory activation of the tissue.

## 3. Discussion

The adipose tissue plays an important role both as an energy reservoir and as an endocrine organ that releases numerous biologically active factors with a broad spectrum of activity [19]. Nevertheless, in the case of excessive energy supply, which we observe during obesity, its activity changes significantly. An excessive accumulation of lipid droplets in obese individuals leads to structural and functional changes in the adipose tissue, which manifest as adipocyte hypertrophy, increased recruitment of immunocompetent cells such as macrophages or lymphocytes, increase in the pro-inflammatory activity and release of cytokines such as TNF-α, MCP-1, and IL-6, imbalance between the release of the anti-inflammatory proteins adiponectin and leptin, and increase in hypoxia and fibrosis [20]. All these changes consequently lead to the development of moderate inflammation accompanying obesity and increased necrosis of adipose tissue cells, which underlie the development of insulin resistance or metabolic syndrome [21,22]. Keeping this in mind, it becomes important to explore the mechanisms of the above phenomenon to find appropriate therapeutic strategies. Therefore, the present study analyzed the role of asprosin in the development and regulation of inflammation by using a cellular model of the co-culture of the main components of the adipose tissue, i.e., adipocytes and macrophages.

The results of the present study showed that asprosin, which is mainly a fasting-induced, glucogenic, and orexigenic adipokine that regulates the body’s energy homeostasis, is also involved in modulating the inflammatory response in hypertrophic adipose tissue. The mechanisms of the development of moderate obesity-associated inflammation, although well described, have not yet been fully explained, for example, from the perspective of new endogenous factors that could act as potential immunomodulators. According to the current research, asprosin seems to be such a modulator, whose activity depends on the degree of maturity and hypertrophy of the co-culture it affects. According to previous scientific reports, in physiological conditions, the concentration of asprosin ranges from 5 to 10 nM [23]; however, in the state of obesity, its value may increase even 5–10 times. In the current work, the effects of both physiological (10 nM) and pathological concentrations of asprosin accompanying obesity (50 and 100 nM) were investigated. Our study used three time points of adipocyte differentiation and maturation, wherein after 21 days of full differentiation, hypertrophic adipocytes corresponding to those observed in obese individuals were obtained. It was observed that by acting on co-cultures on the 21st day of incubation, asprosin in high concentration significantly increased both the expression and the release of crucial pro-inflammatory cytokines such as TNF-α, IL-1β, IL-6. and HMGB1, both from adipocytes and from macrophages. The increase in the concentration of these cytokines is observed in the state of moderate obesity-related inflammation, and their activity is associated with the development of, for example, insulin resistance [21,22]. At the end of last year, Shabir et al. [24] showed that asprosin activates THP-1 macrophages to release TNF-a, IL-12, IL-8, and IL-1b, which may be at least partly related to the TLR4 pathway and NF-kB activation. In turn, the protective effect of asprosin on macrophages was noticed by Zou et al. [25] in an artheriosclerosis model. They observed a reduction in both the accumulation of lipid droplets by macrophages and the formation of macrophage foam cells that was associated with the p38/Elk-1 pathway activation. In our study, it was also observed that high concentration of asprosin increased the migration of macrophages to the supernatant from adipocyte cultures, which was related to the increase in the release of the chemokine MCP-1, the main chemotactic factor for macrophages. This condition may potentially predispose to an increased influx of macrophages to the adipose tissue and, consequently, to an enhanced pro-inflammatory activity. Attention is also drawn to the increase in the release of the highly pro-inflammatory alarmin HMGB1, which in clinical conditions is associated with the development of septic shock [26], and whose elevated level is observed in obese individuals [27]. This alarmin secondarily enhances pro-inflammatory activation through the TLR4/NF-kB downstream pathway activation and the excessive release of pro-inflammatory TNF-α, IL-1β, IL-6 from adipose tissue cells [28]. As reported by Zhang et al. [29], HMGB1 plays a key role in the development of obesity-related inflammation and insulin resistance [30]. It should be noted that HMGB1 can be released passively from damaged, necrotic adipocyte cells [31] and can also be induced, for example, by an increase in the release of pro-inflammatory TNF-α [29], which was observed in the current study. The increase in necrosis is a state typical of the hypertrophic adipose tissue observed in the course of obesity. Our present research showed that asprosin appears to exacerbate the pathological state of moderate inflammation in highly hypertrophic adipose tissue. It also indicates that asprosin may be a target of obesity therapy, not only because of its stimulating effect on food intake but also because of its potentially pro-inflammatory effect. An interesting question seems to be whether the use of asprosin antibodies, apart from the effect of reducing food intake as a result of lowering the concentration of asprosin (as shown by [1]), could have the effect of reducing the severity of obesity-related inflammation. This aspect needs to be explored in future studies.

The effect mentioned above was not observed when the co-cultures were incubated with asprosin at earlier time points, i.e., on the 7th and 14th day of cell maturation. This finding may suggest that the activity of asprosin as a factor promoting inflammatory activation is related to hypertrophic adipocytes typically observed in obesity as a pathophysiological state. Nevertheless, it cannot be ignored that asprosin, acting at earlier time points, reduced the pro-inflammatory activation, thereby exerting a protective effect. This aspect should be investigated in more detail in future studies. The protective effect of asprosin was already demonstrated in mesenchymal stromal cells used in the treatment of myocardial infarction (MI) [32], in cardiomyocytes in hypoxia state [33], and in cardiac microvascular endothelial cells [34], and, hence, asprosin is considered a potential cardioprotective agent. The protective effect of asprosin was found to be associated with a reduction in free radicals resulting from the increase in the expression of antioxidant enzymes, namely, SOD-2, and a reduction in apoptosis by activating the ERK1/2 and PI3K/AKT pathways [32]. Keeping this in mind, it can be seen that the action of asprosin is not universal and depends on the type of cells as well as on the metabolic and physiological state. This is especially apparent in the case of increased damage and apoptosis of pancreatic cells in obese individuals. As asprosin is a recently discovered adipokine, our knowledge of its mechanisms of action is limited, and further research is needed to determine its significance in both physiological and pathophysiological states.

## 4. Materials and Methods

### 4.1. Culture of Murine Cell Lines

The study was conducted on the mouse monocyte–macrophage RAW 264.7 cells and 3T3 L1 preadipocytes differentiated to adipocytes. The 3T3 L1 cell line was kindly provided by Professor Alicja Jozkowicz from the Department of Medical Biology, Jagiellonian University, while the RAW 264.7 cell line was purchased from the European Type Culture Collection (ECACC, Sigma-Aldrich, St. Louis, MO, USA). Both cell lines were tested to be mycoplasma-free. The preadipocytes were differentiated to mature adipocyte using a standard protocol described previously [35]. Briefly, the cells were grown until confluence under standard conditions (37 °C, 5% CO_2_) in DMEM medium supplemented with a 1% antibiotic solution and 10% calf serum. Two days after reaching confluence, the growth medium was replaced with a differentiation medium for the next 2 days (DMEM with 10% fetal bovine serum [FBS], 0.5 mM isobutylmethylxanthine [IBMX], and 0.25 µM dexamethasone). Subsequently, the cells were incubated in DMEM containing 10% FBS and 1 µg/mLof insulin until the end of the 3 week differentiation process. Adipocyte differentiation was evaluated by Oil-Red-O staining. 

To investigate the effects of asprosin on the development of moderate obesity-related inflammation, a co-culture of adipocytes and macrophages, two major adipose tissue populations, was used. To consider the influence of the differentiation stage and maturity of the adipocytes as well as the deposition of lipid droplets, the assessment was performed after 7, 14, and 21 days of differentiation. For the co-culture model, the adipocytes at the various stages of differentiation were kept in the lower parts of 12-well plates, while RAW 264.7 cells were cultured in plate inserts (0.4 µm pore size). The cells were treated with different concentrations of asprosin (0–100 nM) for 24 h. Then, to induce a state of mild inflammation, the co-cultures were stimulated with lipopolysaccharide (LPS; 100 ng/mL; Escherichia coli, serotype 0111: B4; Sigma-Aldrich, St. Louis, MO, USA) for the next 6 h (gene expression studies) or 24 h (protein release studies). During the incubation, 3T3 L1 and RAW 264.7 cells were cultured under standard conditions (37 °C, 5% CO_2_). Fresh cells were used for cytometric assessment and RNA isolation. The supernatants and cell pellets were frozen at −60 °C for quantification of the protein levels.

### 4.2. Cell Activity and Viability

Overall cell viability and activity were measured using a commercial CellTiter kit (Promega, Madison, WI, USA) by assaying the reduction of [3-(4,5-dimethylthiazol-2-yl)-5-(3-carboxymethoxyphenyl)-2-(4-sulfophenyl)-2H-tetrazolium by active mitochondria in the tested cells. The tests were performed according to the manufacturer’s instructions by using a spectrophotometer (Expert Plus, ASYS/Hitech). 

### 4.3. Real-Time PCR Analysis of Gene Expression

Total RNA was purified from 3T3 L1 adipocytes and RAW264.7 macrophages cultured in four separate repetitions for each batch of the experiment. The RNeasy Plus Mini Kit with elimination of genomic DNA was used for RNA extraction. The RNA concentration and quality were assessed using a NanoDrop 2000 spectrophotometer (Thermo Fisher Scientific, Rockford, IL, USA). The High-Capacity cDNA Reverse Transcription kit was used for the reverse transcription of mRNA to cDNA. Real-time PCR gene expression analysis of TNF-α, IL-1β, IL-6, MCP-1, HMGB1, adiponectin, and leptin was performed using the StepOne system from Applied Biosystems and TaqMan primers. Glucose-6-phosphate dehydrogenase (GAPDH) was used as a housekeeping gene for normalizing the amounts of cDNA. Changes in the gene expression level were calculated based on the 2^−∆∆Ct^ algorithm.

### 4.4. Macrophage Migration

To evaluate the effect of asprosin on the release of chemotactic factors responsible for the recruitment of immunocompetent cells, a 48-well microchemotaxis chamber (Neuro Probe) and the RAW 264.7 macrophage cell line were used. The test was performed according to the manufacturer’s protocol. N-formyl-L-methionyl-L-leucyl-phenylalanine (fMLP) was used as a chemoattractant agent in the positive control sample, and DMEM medium was used as the negative control. The chamber was incubated for 45 min under standard culture conditions. The membrane was subsequently washed in PBS and stained. The macrophages that had migrated to the lower side of the membrane were counted in four microscopic fields in each well. The results are expressed as the mean numbers of migratory macrophages per well.

### 4.5. Determination of Cytokine/Chemokine Release

The cytokine levels in the supernatants collected after 24 h of incubation with or without LPS were quantified using a commercial Elisa kit. The concentrations of TNF-α, IL-1β, IL-6, MCP-1, HMGB1, adiponectin, and leptin were determined according to the manufacturer’s instructions and analyzed using an Expert Plus spectrophotometer (ASYS/Hitech, Eugendorf, Austria).

### 4.6. Statistical Analysis

The data were tested for normality of distribution, and the differences among the groups were determined using the Duncan’s new multiple range test 3.1. All data are expressed as mean ± standard deviation (S.D.) with the level of statistical significance (p) set at 0.05.

## 5. Conclusions

This study for the first time assessed the effect of asprosin on the inflammatory activation typical of an obesity-related condition using a co-culture of adipocytes and macrophages. This study allowed us to determine whether the inflammation associated with obesity may be at least in part related to the activity of asprosin, the concentration of which increases in people with obesity. On the basis of the obtained results, it can be concluded that asprosin, as a biomolecule released from the adipose tissue of obese individuals, can be considered an immunomodulatory factor with a potential pro-inflammatory action. Asprosin might not only regulate food intake but also be associated with the pathomechanism of obesity-related diseases through the development of moderate obesity-related inflammation.

## Data Availability

The data in this article will be shared on reasonable request to the corresponding author.
